# Contrasting Effects of Platelet GPVI Deletion Versus Syk Inhibition on Mouse Jugular Vein Puncture Wound Structure

**DOI:** 10.3390/ijms26094294

**Published:** 2025-05-01

**Authors:** Irina D. Pokrovskaya, Kelly K. Ball, Michael W. Webb, Smita Joshi, Sung W. Rhee, Jerry Ware, Brian Storrie

**Affiliations:** 1 Department of Physiology and Cell Biology, College of Medicine, University of Arkansas for Medical Sciences, Little Rock, AR 77205, USA; 2 Department of Molecular and Cellular Biochemistry, College of Medicine, University of Kentucky, Lexington, KY 40536, USA; 3 Department of Pharmacology and Toxicology, College of Medicine, University of Arkansas for Medical Sciences, Little Rock, AR 72205, USA

**Keywords:** GPVI, protein domains, Syk inhibitor, interaction, activation, puncture wound, mouse

## Abstract

Platelet glycoprotein (GP)VI is a transmembrane protein that was originally characterized as a collagen receptor supporting platelet adhesion and activation through its association with the Fc receptor γ-chain (FcRγ). The FcRγ subunit contains immunoreceptor tyrosine-based activation motifs (ITAMs) that recruit and activate Syk (spleen tyrosine kinase), a key player in intracellular signaling pathways. The absence or dysfunction of GPVI produces a mild bleeding defect in humans like the impaired hemostasis reported in the murine knockout. Here, we took an ultrastructure approach to examine the impact of ligand binding to GPVI versus the downstream pharmacologic inhibition of the GPVI-dependent ITAM signaling pathway. Clots were generated for analysis following a puncture wound in the mouse external jugular vein. Images were obtained using mice genetically missing GPVI and mice pretreated with the Syk inhibitor, BI 1002494. Our study was designed to test the hypothesis that the predominant contribution of GPVI to hemostasis is mediated by a Syk-dependent signaling cascade. If true, the clot structure observed with a Syk inhibitor versus the GPVI knockout would be similar. If the extracellular domains of the protein had a Syk-independent platelet adhesion role, then significant comparative differences in the thrombus structure would be expected. Our results clearly indicate an important, Syk-independent role of the GPVI extracellular domain in the adherence of platelets within the intravascular crown of a growing venous clot, a site distant from exposed collagen-rich adventitia. In striking contrast, the adventitial proximal role of GPVI was Syk-dependent, with the GPVI knockout and Syk inhibitor giving the same, limited structural outcome of collagen-proximal platelet cytosol loss and a thinned extravascular cap. Consistent with the lesser role of Syk-dependent processes on the thrombus structure, the Syk inhibitor had no detectable effect on jugular puncture wound bleeding times, while the knockout had a statistically significant, but modest effect on bleeding time. Based on this contrast, we suggest that Syk inhibition may be the more selective approach to modulating the role of GPVI in occlusive clotting.

## 1. Introduction

Platelet glycoprotein VI (GPVI) is both an important platelet plasma membrane receptor protein and an important platelet adhesion protein (for review, see [1,2]). It consists of two extracellular ligand-binding Ig domains, a stalk domain, a single transmembrane domain, and a cytoplasmic domain tail containing calmodulin and Src-kinase-binding sites [2]. Its signaling activity is dependent on the association with Fc receptor γ-chain, which has an immunoreceptor tyrosine-based activation motif (ITAM) [3,4,5]. Ligand binding, for example, collagen or collagen-related peptides, to GPVI leads to Src-kinase-dependent phosphorylation, Syk kinase activation [3,4], and initiation of an intraplatelet signaling cascade, which stimulates platelet aggregation and secretion. In addition to collagen, other GPVI ligands include fibrinogen/fibrin, laminin, and other extracellular matrix proteins [2]. A second platelet ITAM receptor is the C-type lectin receptor 2 (CLEC-2) whose major naturally occurring ligand is podoplanin. In the case of CLEC2, a partial (hem) ITAM motif is directly included in the cytoplasmic tail of the receptor [for review, see, [6,7]. In mice, these are the two ITAM motif receptors. In humans, there is a third.

Functionally, GPVI has important roles in hemostasis and thrombosis. Upon vascular injury or plaque rupture, GPVI interacts with exposed subendothelial extracellular matrix components, principally collagen [8,9,10,11], and initiates a response likely restricted to the immediate damage site. Binding to more recently discovered ligands such as fibrinogen/fibrin for review (see [2]) likely extends more generally throughout the thrombus formation process and could have a greater contribution to the activation of integrin mediated platelet aggregation. Experimentally, knockout of GPVI in mice has a mild effect on bleeding cessation, but a more significant effect on the formation of an occlusive clot, e.g., [12]. One interpretation of this outcome is that GPVI fibrinogen/fibrin interaction is a strong factor in the propagation of platelet aggregation across the multiple hundreds of micron lumen of an artery. In brief, based on these outcomes and speculations, GPVI has become a prime therapeutic target for selective modulation of thrombosis versus hemostasis.

In the present study, we have taken an ultrastructure approach to reexamine the relative contribution to hemostasis of GPVI as an adhesion protein versus a signaling receptor. We used a mouse puncture wound structure model and compared the effect on thrombus ultrastructure of knocking out the protein and hence eliminating both its adhesion and signaling functions versus electively inhibiting its signaling function only through pretreatment of mice with the Syk inhibitor, Bi 1002494 [13]. Our hypothesis was that if the predominant contribution of GPVI to hemostasis was mediated by a Syk-dependent signaling cascade, then the structural effect of a Syk inhibitor and a GPVI knockout would be same. If the extracellular domains of the protein had an important platelet adhesion role, then significant comparative differences in thrombus structure would be expected. Our experimental outcomes strongly indicate that GPVI extracellular domains play a crucial role in how tight the adherence of platelets within the intravascular crown of the jugular vein puncture is.

## 2. Results

**Figure 1:**
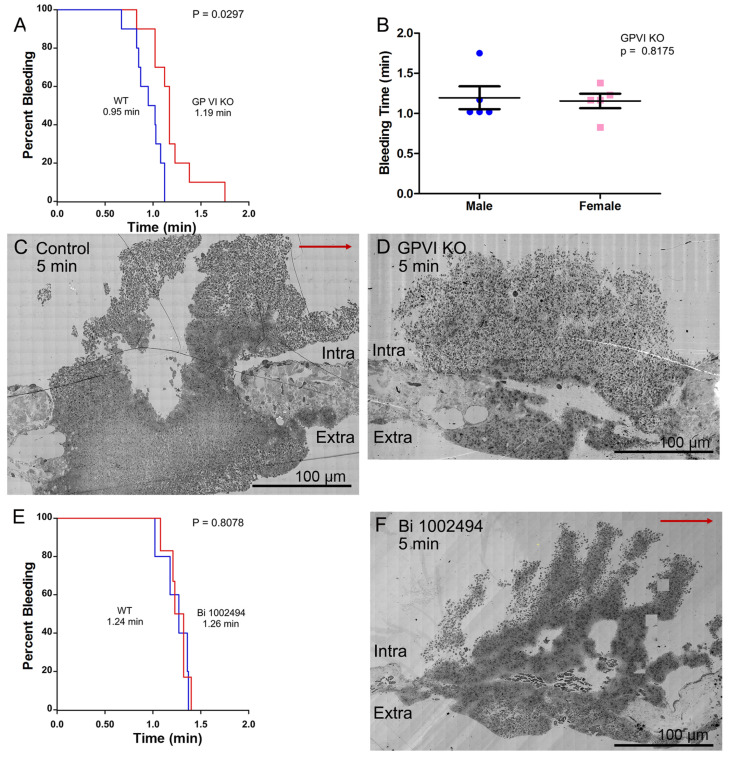
Bleeding effects and thrombus structure at 5 min post puncture wounding. All thrombi, darker, platelet-rich structures (**C**,**D**,**F**), are arranged as intravascular side to top, extravascular side to bottom, flow left to right. All bleeding times (**A**,**B**,**E**) are for the jugular vein puncture wound model. (**A**,**E**) are presented as Kaplan–Meier plots where the Y axis; percent bleeding is the % of that animal population. The red arrow in C indicates the direction of blood flow. The diredtion is the same in D and F, (**C**–**E**) are shown at 2% zoom of the original WA-TEMs. In total, 44 mice were used and approximately 1800 individual electron microscope frames, 4 K by 4 K pixels were taken and then montaged together to generate the images shown.

Consistent with previous research [12], the deletion of GPVI produces a mild bleeding defect with a 25% increase in time to bleeding cessation in a mouse jugular vein puncture wound model (Figure 1A Kaplan–Meier plot, B). The increased bleeding time was accompanied by two significant changes in thrombus ultrastructure. As shown in Figure 1C, wild-type, versus Figure 1D, GPVI knockout, the GPVI knockout thrombus at 5 min post puncture was altered in both the structure of the intravascular thrombus crown and the extravascular thrombus cap, which prevents further bleeding. The tightness of platelet adherence within the crown was visibly decreased. The extravascular cap of the knockout was thin compared to that in the wild type and similar in appearance to that formed in a Syk-inhibitor-treated mouse [13], Bi 1002494, 5 min post puncture, as shown in Figure 1F. The intravascular thrombus crown in the inhibitor-treated mouse was like that in the wild type, indicating normal platelet adhesion. Consistent with the more normal thrombus structure in Bi-1002494-treated mice, bleeding time in the Syk-inhibitor-treated mice was normal (Figure 1E, Kaplan–Meier plot). Overall, these differences indicate a non-signaling, Syk-independent, adherence role for the extracellular domains of GPVI in the formation of an intravascular thrombus crown.

**Figure 2:**
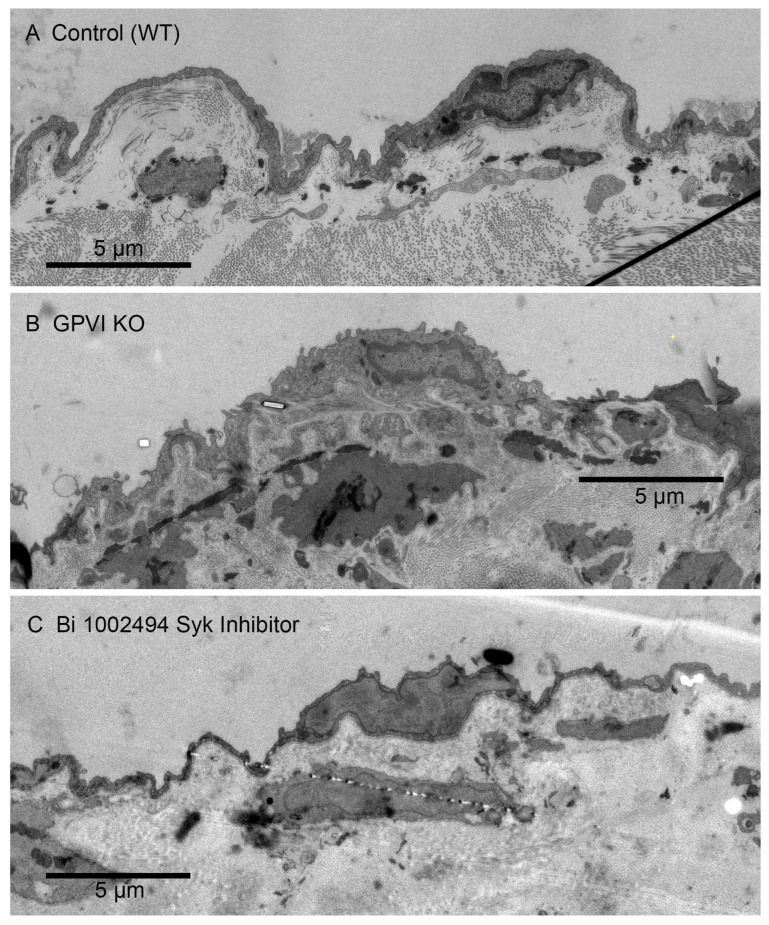
Appearance of the endothelial cell layer and associated jugular vein vessel wall components in Control (WT, **A**), GPVI knockout (KO, **B**) and Bi-1002494-treated mice (**C**). Five min post puncture wounding. In all three cases, the vessel wall segment shown is an undamaged area close to the wound hole. Appearance is similar in all three cases. The intravascular space is to the top in each image. Images are shown at a 25% zoom of the original WA-TEMs. Three individual mice were used. Again, multiple electron microscope frames were montaged.

As controls for possible significant non-platelet effects of the Syk inhibitor administration and its efficacy in vivo, we carried out multiple additional tests. The first was to test if the gavage protocol used for drug treatment had significant effects on the arterial occlusion induced. Bi 1002494 is a known, preferential inhibitor of occlusive clotting [13]. In a ferric-chloride-induced occlusive clotting assay, we found that two rounds of mock, water gavages had no effect on occlusion time (3–3.5 min) or the formed clots as detected by intravital video microscopy. In brief, no gavage effect was detected. Second, as expected [13], the gavage-administered Bi 1002494 treatment in parallel experiments inhibited the formation of occlusive thrombi. Third, we tested for off-platelet vascular effects of the standard Bi 1002494 treatment by assessing the effects on the endothelial cell layer and underlying vessel wall. Morphologically, these features were similar in wound distal, jugular vein regions of Control (wild type), GPVI KO and Bi-1002494-inhibitor-treated mice (Figure 2). There was no visible evidence for brekas in the endothelial plasma membrane nor any evidence for loss of cytoplasmic contents. Overall, our results indicate that Bi 1002494 treatment produced platelet-specific outcomes reflective of in vivo efficacy. We note that the reported concentration of Bi 1002494 in mice under our gavage conditions is 940 nmol/L, 6-fold higher than that required to abrogate collagen-related peptide responses of wild-type platelets in vitro [13].

**Figure 3:**
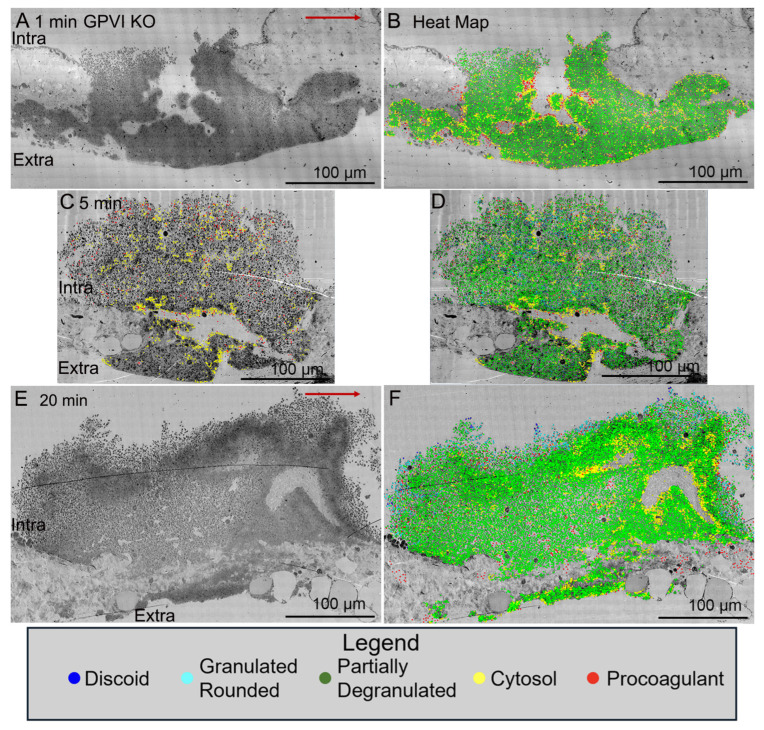
Kinetics of thrombus formation, GPVI knockout mice, raw and platelet activation state mapped (red/yellow high activation and blue discoid-shaped platelets; cyan, green in between, based on morphology, granule presence, cytoplasm). Thrombi, darker, platelet-rich structures, are arranged as intravascular side to top, extravascular side to bottom, Arrow, blood flow within the vein left to right. (**A**,**B**). 1 min time point, raw and platelet activation state annotated images. (**E**,**F**). 20 min time point, raw and platelet activation state annotated images. An example of high-activation platelets within vault region of a 5 min thrombus is emphasized in (**C**) by heat mapping only high-activation platelets (yellow and red) compared to (**D**), five color heat mapping of all activation states. The red arrows indicate the direction of blood flow. Images are shown at 2% zoom of the original WA-TEMs. Three individual mice were used and approximately 1800 individual electron microscope frames, 4 K by 4 K pixels were taken and then montaged together to generate the images shown.

Significantly, when examined as a time series, jugular vein puncture wound thrombi from GPVI knockout mice appeared more abnormal in structural organization at late versus early time points, 20 min versus 5 min, or 1 min. For an unannotated Control, 5 min (WT), please see Figure 1C. As shown in Figure 3A, a 1 min knockout thrombus was composed primarily of tightly adherent platelets with a thick extravascular cap, while at later time points, 5 min (Figure 3C, Figure S1, N = 3 in total) and 20 min (Figure 3E), the thrombi formed had progressively thinner extravascular caps and a decreased incidence of tightly adherent platelets in the intravascular crown. Counter intuitively, this outcome suggests that the collagen-binding function of GPVI is of limited importance to platelet adherence during the initial steps in thrombus formation. If anything, these results indicate that the fibrinogen/fibrin-binding activity of the extracellular D1/D2 domains of GPVI becomes increasingly important during thrombus formation/maturation. Two-dimensional heat mapping of platelet activation state indicated a concentration of highly activated platelets, organelle-free cytoplasm (examples demarcated by yellow dots) and cytoplasm-free, membrane ghosts (procoagulant platelets, red dots) on the periphery of intra-thrombus vaults and “fjords”, red dots). Interestingly, knockout intra-thrombus vaults appeared to be enriched in apparent procoagulant platelets at 5 min post puncture wounding (for example, Figure 3C, see also 5 color frames). A concentration of highly activated platelets to the peripheral surfaces of intra-thrombus vaults has been noted previously [14]. For I min, 5 min and 20 min annotated Control (WT) images, please see [14].

**Figure 4:**
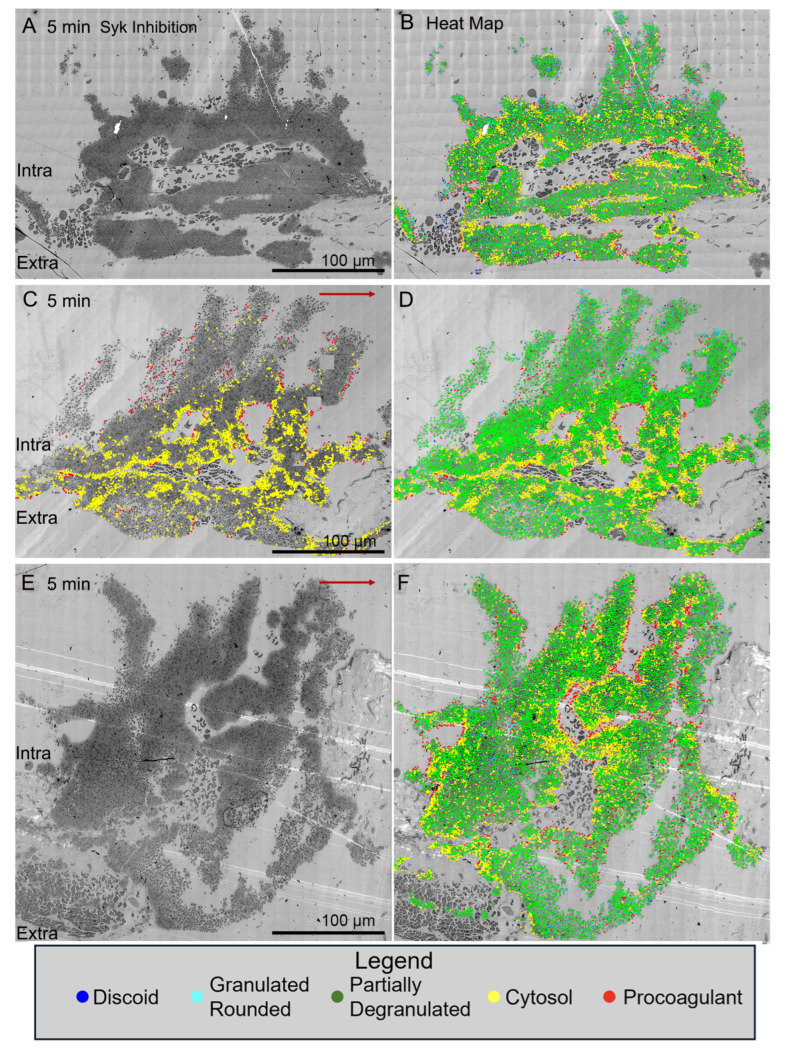
Effect of SYK inhibition on thrombus morphology at 5 min post puncture raw and platelet activation state mapped (red/yellow high activation and blue discoid-shaped platelets; cyan, green in between, based on morphology, granule presence, cytoplasm). Thrombi, darker, platelet-rich structures, are arranged as intravascular side to top, extravascular side to bottom, blood flow within the vein left to right. (**A**,**B**). 1 min time point, raw and platelet activation state annotated images. (**E**,**F**). 20 min time point, raw and platelet activation state annotated images An example of high-activation platelets within vault region of a 5 min thrombus is emphasized in **C** by heat mapping only high-activation platelets (yellow and red) compared to **D**, five color heat mapping of all activation states. The red arrows indicate the direction of blood flow. Images are shown at 2% zoom of the original WA-TEMs. Three individual mice were used and approximately 1800 individual electron microscope frames, 4 K by 4 K pixels were taken and then montaged together to generate the images shown.

In contrast, all three examples of jugular puncture wound thrombus formation in Syk-inhibitor-treated mice showed a more normal formation of an intravascular thrombus crown with extensive vaulting at 5 min post puncture (Figure 4). The platelet-rich columns delimiting the vaults were mostly rich in tightly adherent platelets with some loosely adherent platelets coating the blood stream proximate surfaces of the columns. As noted in Figure 1F, the extravascular cap producing bleeding cessation was thin with some loosely adherent platelets apparent in these Syk-treated mice. Heat mapping platelet activation state in the thrombi revealed an absence of free-procoagulant platelets (red dots) within vaults, as in the case of GPVI knockout mice (Figure 3C versus Figure 4B,D,F).

**Figure 5:**
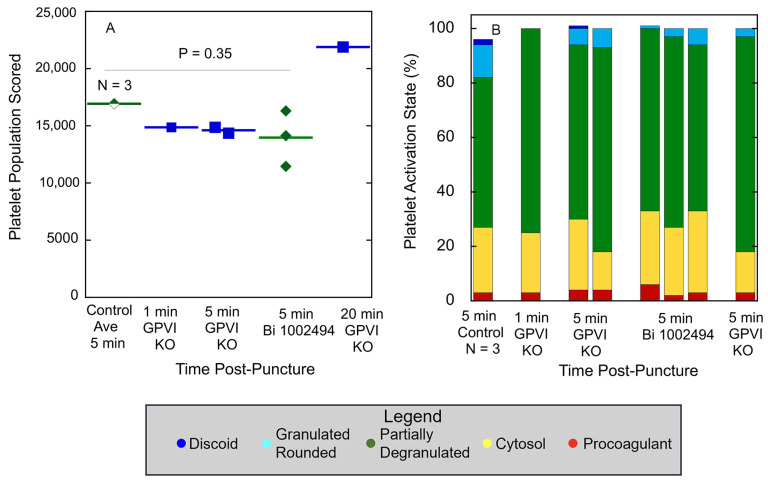
GPVI knockout and SYK inhibitor treatment have a similar effect on thrombus size and distribution of thrombus-recruited platelets between different morphologically scored activation states. (**A**) Platelet abundance in mid-thrombus WA-TEM images, (**B**) Distribution between activation states, WA-TEM images/outcomes are compared to 5 min post puncture, no-drug-treated jugular vein thrombi, as a WT-Control. In (**B**), the columns do not always add up to 100 because of rounding effects. *p* value for Control versus Bi 1002494 samples was calculated using Student’s *t*-test.

Quantitatively, as shown in Figure 5A, platelet abundance in time-matched, 5 min puncture wound thrombi from GPVI knockout, Syk-inhibitor-treated, and Control mice was similar. The size of the 20 min thrombus example in the GPVI knockout was almost 22,000 platelets, a value almost 50% higher than that found at earlier times points. This value is almost twice that reported earlier for Control, wild-type C57Bl/6 mice [14]. In brief, the data provide quantitative evidence that GPVI is not required for platelet recruitment to a forming, hemostatic, jugular vein, puncture wound thrombus. Scoring of the individual platelet profile activation state based on morphological criteria showed little to no difference in distribution between the various states based on how GPVI function was disrupted (Table 1 and Table 2; Figure 5B), an indication that the activation state in more dependent on Syk kinase signaling than platelet adherence is. In comparison to control, wild-type C57Bl/6 jugular puncture wound thrombi (Figure 5B), the distribution of the platelet activation state as displayed in GPVI-function-disrupted thrombi was shifted towards partially degranulated platelets (green). In a final quantitative comparison, we compared platelet neighbor pairings across the two different GPVI perturbant conditions. In these comparisons, platelet pairing within a 2 mm radius was determined. As shown in Table 1 and Table 2, like paired with like, more than predicted by chance, confirming the null hypothesis; partially degranulated platelets (green) had a high probability of pairing with one another, a tendency that was more marked than in wild-type thrombi [14].

**Figure 6:**
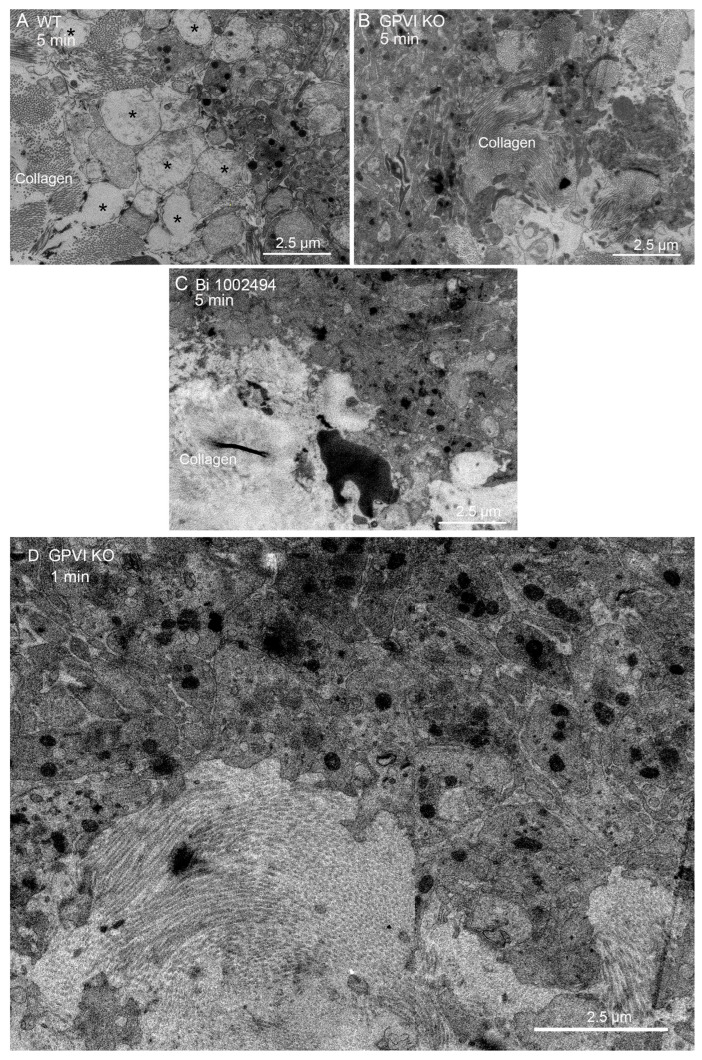
Both GPVI knockout and Bi 1002494 treatment inhibit platelet cytosol loss at the thrombus/collagen-rich adventitia interface. (**A**) WT, 5 min post puncture wounding, asterisks mark examples of platelet cytosol loss. (**B**,**C**) Such loss was not observed with either GPVI perturbation or Bi 1002494 treatment, 5 min post puncture wounding. (**A**) Collagen to left side; (**B**) collagen to right-hand side; and (**C**), collagen to lower left. (**D**) One min post puncture wounding. Remaining organelles in the platelet cytoplasm are primarily mitochondria. Note: mitochondrial cisternae are not prominent at this zoom. Collagen shows as bundled fibers or dots depending on orientation within the plane of the section.

Qualitatively, one would expect that any adhesive or signaling role of collagen through binding to GPVI would be most obvious at the platelet collagen adventitial interface. This expectation is confirmed in Figure 6A, where collagen proximal platelets in a 5 min wild-type thrombus were often degranulated and nearly devoid of cytoplasmic contents (see also [15]). However, cytosol loss did not occur in 5 min thrombi from either GPVI knockout mice or Syk-inhibitor-treated mice (Figure 6B,C). In both cases, platelet–platelet adherence appeared normal. Similar results were observed at an early, 1 min time point for the GPVI knockout (Figure 6D). Overall, our results strongly indicate a dependence of platelet activation state on collagen-associated Syk-dependent signaling but provide little to no evidence for collagen-associated platelet–platelet adhesion. Note that dabigatran inhibition of thrombin activity produces a similar inhibition of platelet cytosol loss at the adventitial interface [15]. This was not true with cangrelor, a direct P2Y_12_ inhibitor [15]. These outcomes suggest that cytosol loss is differentially dependent on multiple signaling pathways.

## 3. Discussion

Taking an ultrastructural approach to re-examine the in vivo role of platelet glycoprotein (GP)VI in thrombus structure and bleeding cessation, we found that GPVI knockout had significantly greater effects on bleeding cessation times and mouse, jugular vein, puncture wound thrombus formation, i.e., initial steps in primary hemostasis, than the treatment with the Syk inhibitor Bi 1002494 did [13]. In agreement with previous results in other bleeding assays, GPVI knockout gave a mild, but significant prolongation of bleeding times, while the Syk inhibitor had no effect on bleeding cessation time. We attribute these differences to the greater effect of the GPVI knockout on the thrombus structure. Both the protein knockout and Bi 1002494 inhibited cytoplasm loss from platelets at the collagen-rich adventitia interface, a near-terminal step in a platelet activation sequence, indicating a Syk dependence. Significantly, this inhibition had no effect on bleeding time in the Syk treatment case, indicating that this activation state is not required for bleeding cessation. Moreover, neither perturbation had any effect on platelet–platelet adherence at the adventitial interface. Importantly, both GPVI knockout and Bi 1002494 yielded at 5 min post puncture a thinned and less tightly adherent extravascular cap, the structural element producing bleeding cessation [15], indicating a greater sensitivity of the ultrastructural assay than the more common bleeding time assay. Importantly, the wound thrombus formed in the GPVI knockout appeared to be much more porous, containing extensive intravascular areas of loosely adherent platelets that were found at 5 and 20 min post puncture to protrude into the extravascular bleeding cessation cap. We project that the steps leading to this outcome are the cause of the longer bleeding times in the GPVI knockout mouse.

**Figure 7:**
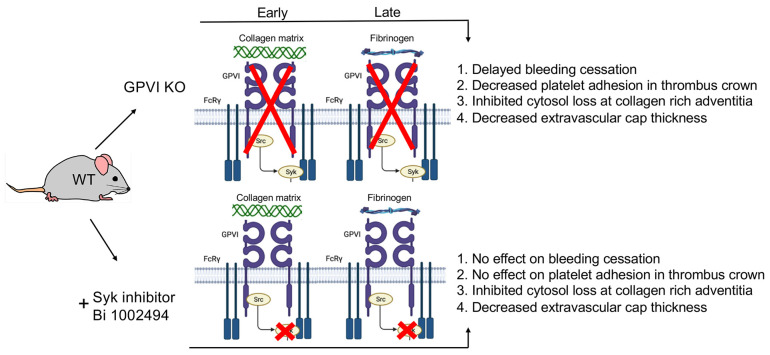
Data based model of the role of GPVI in the formation of a vein puncture wound thrombus. Four traits were scored on the basis of morphology in montaged wide-area transmission electron micrographs (WA-TEM). Note that there were no Syk-inhibitor-detected traits that were not shared with the GPVI KO. The large red XX’s indicate that the gene knockout affects the whole protein. The small redc’sred x’s indicate that the drug treatment only affects reactions at the tail of the protein.

The comparative structural outcomes provide direct visual evidence GPVI acts as both a signaling protein and a late acting, major adhesive protein in normal hemostasis. Contrary to expectation for a protein originally discovered as the major platelet collagen receptor [8,9,10,11]. We found that the adhesive effect of GPVI became prominent not at the early time point of 1 min but rather later, 5 and 20 min, and was most pronounced intravascularly in the thrombus crown, a site distant from the collagen-rich adventitia. We attribute this outcome to the importance of the fibrinogen/fibrin binding activity of the extracellular D1/D2 domains of GPVI for review, see [2]. This outcome lends further support to the longstanding dogma for a dominant role of the collagen/von Willebrand factor/platelet glycoprotein Ib-IX axis in primary platelet adhesion [16,17,18,19,20,21,22]. The structural consequences of Syk-dependent inhibition were chiefly manifested on the structure of the platelet-rich extravascular thrombus cap and had no effect on bleeding time. This minor outcome is a strong indicator of the limited role of Syk-dependent signaling in hemostasis. In brief, our studies suggest that the major adhesive role of GPVI in thrombus formation is as a fibrinogen/fibrin binder rather than as a collagen binder. For an overview model of our ultrastructural outcomes, see Figure 7. In part, this model is proposed to reflect the fact we detected no Syk inhibitor morphological outcomes that were not shared with the GPVI KO, an indicator that GPVI is the dominant target of Syk inhibition in mouse platelets.

In conclusion, platelet glycoprotein VI has been long considered to be a therapeutic target for controlling thrombosis because of its mild effects on hemostasis [12,23,24,25,26,27]. Our ultrastructural outcomes indicate that targeting the extracellular domains of GPVI could well have more consequences for hemostasis than inhibiting GPVI’s Syk-dependent intracellular signaling role. Syk targeting could well be the safer route, albeit of untested ultrastructural consequences for occlusive clotting.

## 4. Materials and Methods

### 4.1. Mice and Reagents

All animal usage was approved by the relevant local Institutional Animal Care and Use Committees. Wild-type C57BL/6 or GPVI^−/−^ male and female mice ([12], 8–12 weeks old) were used in equal numbers across the individual datasets. All reagents were of reagent grade and listed previously [27,28,29].

### 4.2. Bi 1002494 Spleen Tyrosine Kinase (Syk) Inhibitor Treatment

The mice were treated with Bi 1002494 Syk inhibitor as described earlier [13]. In brief, Bi1002494 was dissolved in deionized water and administered (100 mg/kg body weight) twice (15 h and 1 h before clot induction) by oral gavage [13]. For gavage controls, animals were gavaged with the equivalent volume of deionized water.

### 4.3. Occlusive Clotting Tests for Gavage Effects and In Vivo Bi 1002494 Treatment Efficacy

The effect of water (Control) and Bi 1002494 inhibitor gavages on ferric chloride induced occlusive clotting was monitored visually using a Zeiss stereo Discovery v12 microscope, Axiocam 305 color camera (Carl Zeiss, White Plains, NY, USA), and blood flow was quantified using a TS420 transit-time perivascular flowmeter and sonic probe (Transonic Systems, Inc., Ithaca, NY, USA). Clotting of the femoral artery was induced using a small piece of Whatman filter paper, grade 4 (~350 µm × ~500 µm), soaked with 0.5 µL of a 10% FeCl_3_ (Fisher Scientific, Waltham, MA, USA) solution prepared immediately before use in deionized water. The filter paper was placed perpendicularly on the artery and left for 3 min. Residual FeCl_3_ was rinsed away with room temperature (RT) saline. Blood flow/clot formation was monitored for 10 min post FeCl_3_ injury and positive/negative clotting was scored at the 10 min time point [30].

### 4.4. Thrombus Preparation and Electron Microscopy [15,28,29]

Jugular vein wounding was carried out with a 30 G needle (312 μm nominal diameter) and thrombi were fixed in situ at 1, 5, and 20 min, post injury, with 4% paraformaldehyde. For WA-TEM, the samples were processed for plastic embedding as previously described and stained with uranyl acetate and lead citrate post-embedding [15,28]. Automatically montaged images were collected at 3.185 nm XY pixel size using SerialEM software (version 3.6, 32-bit) and visualized with 3DMOD software (version 4.9.13). Fine image blending was carried out with eTomo software (version 4.11.12). Image visualization was carried out with IMOD software (version 4.11). iMac Pro computers (MacOs 10.14, 5K display) were used, and the images were displayed at various zoom factors ranging from 2% to 100%. The raw images were as large as 130,000 by 90,000 pixels. All software is currently available from the Mastronarde group at https://www.colorado.edu/mcdb/resources/mastronarde-group (accessed on 22 April 2025).

### 4.5. Datasets

All datasets will be deposited and publicly available as raw images upon acceptance of this manuscript. All mapping analysis, and the resulting quantitative and mapping imagery are unique and original to the present work.

### 4.6. Manually Annotated Platelet Activation State Heat Mapping and Nearest Neighbor Analysis [14]

Montaged WA-TEM images recorded in 24-bit .mrc format were blended into a single image using eTomo (IMOD software package), binned (~5 × 5) in 3DMOD, and converted to 8-bit grayscale images with FIJI software. Platelet activation states were annotated in the binned images. and displayed in 8-bit color using iVision-Mac software (version 4.5.4, 32-bit software, BioVision Technologies, Inc., Exton, Pennsylvania, PA, USA). From the WA-TEM images, the platelets were classified into five groups based on their morphology: discoid (blue); rounded, granulated (cyan); partially degranulated (green); cytosol-rich, degranulated, but containing mitochondria (yellow); and devoid of internal contents (red). On the images, each platelet was scored, marked with a point in its center (centroid) which was given a unique identification number, and its X, Y coordinates were recorded. Platelet scoring was carried out manually by outcome-blinded scorers. The ‘Measure Segments’ command in iVision was used to evaluate the distributions of platelet states in the region surrounding each individual platelet. For each centroid, the state of all adjacent centroids, within a 2 µm radius, was tabulated. This was repeated iteratively for each platelet centroid in each field. The clustering of different platelet classes was calculated using the average numbers of each class associated with a given centroid and expressing that as a percentage of the total for that class. Pairing percentages were tabulated for each class in a series of rows reading from left to right for a given activation with the individual pairings then arranged column-wise. Each individual row adds up to 100% as the full set of pairing for that activation state. Bold-faced, large-font pairing values indicate statistically significant localized co-clustering of that activation state (Table 1 and Table 2). The calculation formulas can be revealed by clicking on the Excel spreadsheet example.

Statistical significance was calculated as shown in the Excel spreadsheet example presented in the Supplemental Materials. In brief, the experimental values and the values expected from the null hypothesis of random platelet pairing were compared and those with a positive Z score value greater than 2.56 were considered significant. For the sake of simplicity, only values with a significant positive score, i.e., greater than expected from random, are highlighted. The effect values (highlighted in purple in the tables) were considered significant if 10 or greater. Between ~11,000 and ~22,000 platelet profiles were evaluated for each image set.

## Figures and Tables

**Table 1:** GPVI KO Thrombi: Platelet Abundance and Platelet Neighbor Pairing.

1 Min GPVI KO: 14,863 Platelets Scored					
State	Blue	Cyan	Green	Yellow	Red
Pop %	0%	0%	75%	22%	3%
Blue with	0%	0%	100%	0%	0%
Cyan with	0%	**5%**	83%	11%	1%
Green with	0%	0%	**83%**	17%	1%
Yellow with	0%	0%	51%	**45%**	**4%**
Red with	0%	0%	17%	**40%**	**43%**
5 min GPVI KO: 14,349 platelets scored					
State	Blue	Cyan	Green	Yellow	Red
Pop %	1%	6%	64%	26%	4%
Blue with	0%	17%	57%	20%	7%
Cyan with	0%	**11%**	**81%**	8%	0%
Green with	0%	4%	**83%**	12%	0%
Yellow with	0%	1%	20%	**79%**	1%
Red with	1%	0%	7%	34%	**58%**
20 min GPVI Knockout: 21,884 platelets scored					
State	Blue	Cyan	Green	Yellow	Red
Pop %	0%	3%	79%	15%	3%
Blue with	**33%**	25%	38%	4%	0%
Cyan with	1%	**32%**	47%	7%	**12%**
Green with	0%	1%	**87%**	12%	1%
Yellow with	0%	0%	51%	**48%**	1%
Red with	0%	**11%**	63%	13%	**14%**
Low to high activation state -----> 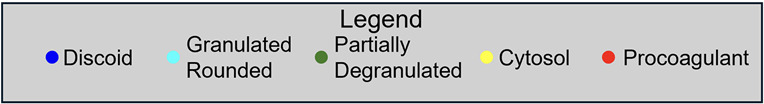					

Bold faced, large type values are statistically positive from the random null hypothesis based on a Z score of 2.56 or greater. Values highlighted in purple have an Effect score of greater than 10.

**Table 2:** Bi 1002494 Thrombi: Platelet Abundance and Platelet Neighbor Pairing.

Example 1. 05 Min Bi 1002494: 16,300 Platelets Scored					
State	Blue	Cyan	Green	Yellow	Red
Pop %	0%	1%	67%	27%	6%
Blue with	0%	0%	0%	0%	0%
Cyan with	0%	**14%**	65%	13%	8%
Green with	0%	1%	**77%**	21%	2%
Yellow with	0%	0%	40%	**57%**	4%
Red with	0%	1%	29%	28%	**42%**
Example 2. 05 min Bi 1002494: 14,127 platelets scored					
State	Blue	Cyan	Green	Yellow	Red
Pop %	0%	3%	70%	25%	2%
Blue with	10%	15%	65%	5%	5%
Cyan with	0%	**14%**	**79%**	5%	1%
Green with	0%	2%	**85%**	12%	1%
Yellow with	0%	0%	29%	**69%**	1%
Red with	0%	2%	36%	28%	**34%**
Example 3. 05 min Bi 1002494: 11,453 platelets scored, perpendicular to flow					
State	Blue	Cyan	Green	Yellow	Red
Pop %	0%	6%	61%	30%	3%
Blue with	**34%**	20%	20%	3%	**23%**
Cyan with	0%	**9%**	**76%**	14%	1%
Green with	0%	6%	**74%**	19%	0%
Yellow with	0%	2%	34%	**63%**	1%
Red with	1%	4%	18%	35%	**41%**
Low to high activation state -----> 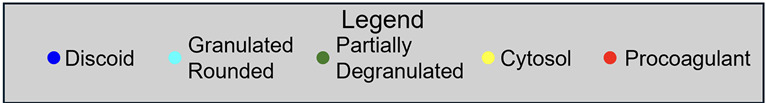					

Bold faced, large type values are statistically positive from the random null hypothesis based on a Z score of 2.56 or greater. Values highlighted in purple have an Effect score of greater than 10.

## Data Availability

All image datasets will be deposited in the Microscopy Public Image Archive (https://www.ebi.ac.uk/empiar) and will be publicly available as raw images upon acceptance of this manuscript. All mapping analysis, and the resulting quantitative analysis, and mapping imagery are unique and original to the present work.

## Supplementary Materials

The following supporting information can be downloaded at: https://www.mdpi.com/article/10.3390/ijms26094294/s1
