# Supplementary material for: Contrasting Effects of Platelet GPVI Deletion Versus Syk Inhibition on Mouse Jugular Vein Puncture Wound Structure
Source: Int J Mol Sci. 2025 May 1;26(9):4294. doi: 10.3390/ijms26094294 (PMC12072639; doi:10.3390/ijms26094294)
Supplement: Supplementary file 1 [file ijms-26-04294-s001.zip › ijms-3571184-supplementary.pdf]

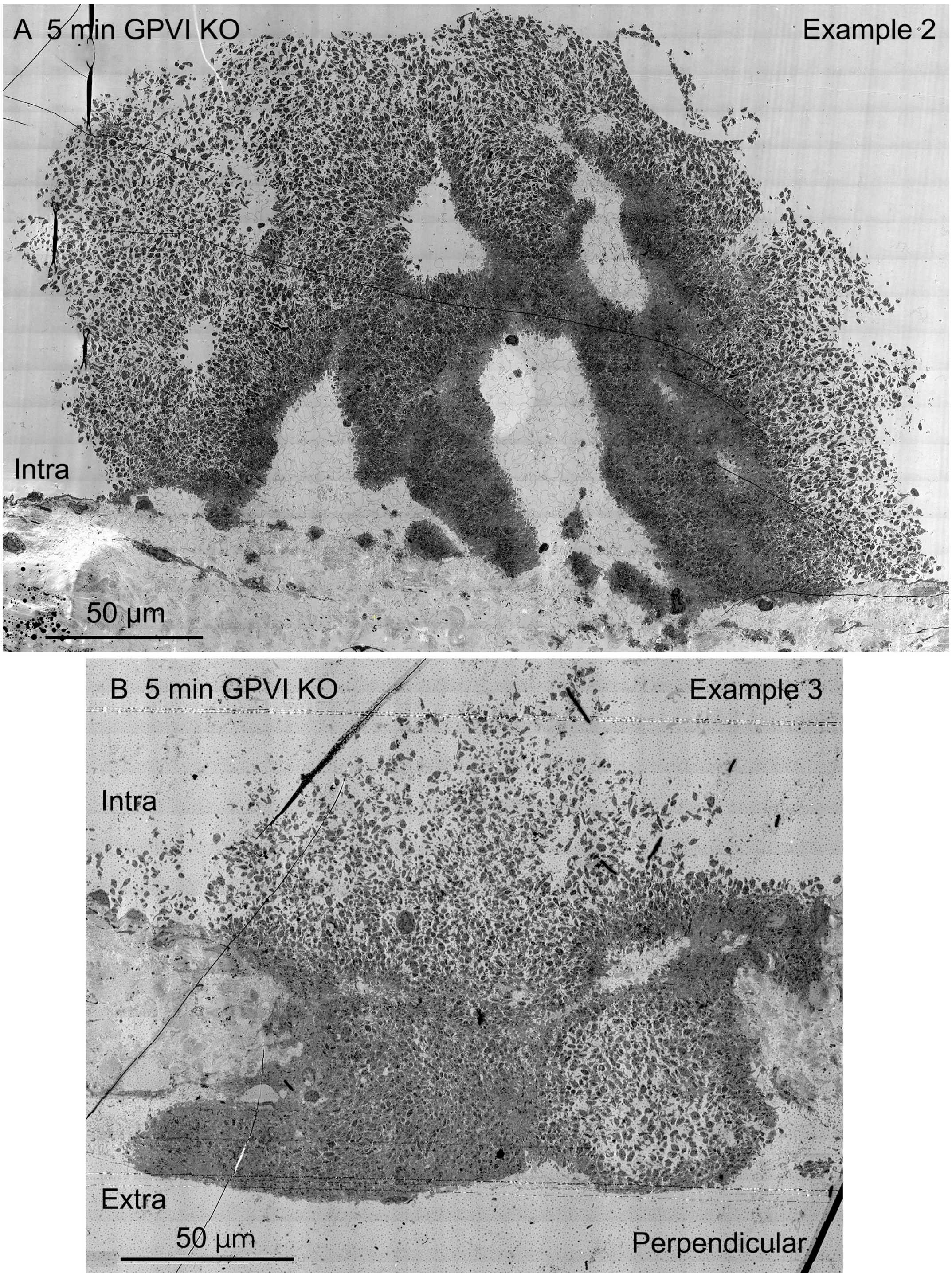

Figure S1. Representative images of two (**A,B**) additional 5-minute GPVI knockout (KO) thrombi. Including the example shown in the main text, a total of **N = 3** GPVI KO thrombi are presented for the 5-minute time point.
